# Association between cognition and color discrimination among Lebanese patients with schizophrenia

**DOI:** 10.1186/s12888-022-04245-y

**Published:** 2022-09-12

**Authors:** Oussama Dahdouh, Tala Solh, Corinne Lahoud, Chadia Haddad, Souheil Hallit

**Affiliations:** 1 Research Department, Psychiatric Hospital of the Cross, Jal Eddib, Lebanon; 2 Faculty of Sciences, Lebanese University, Fanar, Lebanon; 3 School of Medicine and Medical Sciences, Holy Spirit University of Kaslik, P.O. Box 446, Jounieh, Lebanon; 4 INSPECT-LB (Institut National de Santé Publique, d’Épidémiologie Clinique et de Toxicologie-Liban), Beirut, Lebanon; 5 School of Health Sciences, Modern University for Business and Science, Beirut, Lebanon; 6 Psychology Department, College of Humanities, Effat University, Jeddah, 21478 Saudi Arabia

**Keywords:** Color discrimination, Cognitive function, Psychotic symptoms, Schizophrenia

## Abstract

**Background:**

Patients with schizophrenia (SCZ) exhibit poorer color discrimination than normal individuals. Although retinal abnormalities, as well as cortical and subcortical alterations, found in patients with SCZ have been suggested to cause this poor color discrimination, the impact of cognitive impairment remains to be determined. Dopamine (DA) and glutamate (Glu), known to be disrupted in SCZ, are also suggested to play a role in color discrimination. Our objective was to investigate the contribution of cognitive impairment to color discrimination deficits in SCZ and to examine if these deficits are correlated to SCZ symptoms.

**Methods:**

This study includes 127 patients with SCZ between July and September 2021. The participants completed several questionnaires, specifically the Positive and Negative Syndrome Scale (PANSS), the Montreal Cognitive Assessment (MoCA) test, and the Farnsworth D-15 test, to assess the extent of SCZ symptoms, cognition, and color discrimination respectively.

**Results:**

Higher cognition (Beta = − 0.279) was significantly associated with a lower total error score (TES). Moreover, a higher positive PANSS score (Beta = 0.217) was significantly associated with a higher TES. A multinomial regression analysis taking the type of color blindness as the dependent variable showed that female sex (ORa = 5.46) was significantly associated with a certain type of color blindness.

**Conclusion:**

Color discrimination deficits in patients with SCZ may be due to the effect of cognitive impairment and/or SCZ itself.

## Background

Schizophrenia (SCZ) is a complex mental disorder manifested through a variety of positive, negative, and cognitive symptoms [1]. However, recent studies showed that in addition to cognitive dysfunctions, such as memory and attention problems, SCZ is associated with abnormalities in basic sensory processes such as vision and olfaction [2–6]. Schizophrenic patients (SP) usually suffer from deficits in visual perception. These deficits include reduced contrast sensitivity, impaired motion processing and spatial localization, as well as a greater sensitivity to backward masking and color vision impairment [7]. The underlying mechanisms responsible for these deficits could be explained by alterations at the level of the retina and/or at the subcortical and cortical levels [5, 8].

### Visual abnormalities and color vision in SCZ

Color blindness happens when one or more of the light-sensitive photoreceptors found in the retina, the cone color cells, are either absent, not working, or malfunctioning by detecting a different color than normal [7]. Depending on the photoreceptors affected, there are three types of color blindness. The impairment of blue light photoreceptors leads to a tritan color blindness. Green light photoreceptors could also be affected causing deutan color blindness while protan color blindness occurs due to problems in the red light photoreceptors. The type of affected photoreceptors also determines the deficient hue axis. The deficient hue axis represents a region on a color wheel where the patient has problems discriminating among colours. There are three hue axes including the blue-hue axis, the green-hue axis and the red-hue axis [9].

In standard cases, color blindness is usually congenital. However, when color vision problems occur later in life, they usually result from disease, trauma, drug toxicity, metabolic disease, or vascular disease [7]. Compared to other visual deficits, color vision in SCZ has obtained little investigation [10].

### Retinal abnormalities and color vision

It is crucial to study retinal abnormalities in SCZ as many conditions (Parkinson’s disease (PD), retinal dystrophies…) involving these abnormalities share similar visual impairments with SCZ (contrast sensitivity, color perception…) [8]. Ophthalmologic studies have found various visual abnormalities in the primary constituents of the optic pathway in SP. These abnormalities could be structural or functional and cause the disruptions in visual processing experienced in SCZ. Studies employing optical coherence tomography (OCT) in SCZ uniformly show evidence-based structural retinal pathology. However, the location of the pathologies often differs across these studies [8]. Such pathologies encompass retinal nerve fiber layer (RNFL) thinning caused by loss of retinal ganglion cells (RGC). The axons of the RGC form the RNFL that exits the retina as the optic nerve and targets the lateral geniculate nucleus (LGN). This is where it provides input to the magnocellular (M) and parvocellular (P) pathways [3]. RGC loss could be due to excessive glutamate (Glu), a crucial neurotransmitter in the retina. When in excess, Glu could exert a toxic effect leading to neurodegeneration and RGC destruction in SCZ [11, 12]. It is considered that RGC loss may cause perturbations of visual processing, such as abnormal color perception and contrast sensitivity [11, 13].

Macular thinning, particularly thinning of the fovea and its surrounding tissue, and macular volume reduction are also observed in SCZ [3, 8]. In that regard, it is important to note that cones are concentrated in the fovea [14]. Electroretinography (ERG) studies in SCZ indicate decreased photoreceptors activity. Using a portable ERG device, Demmin et al. found photoreceptor and bipolar cell activity to be reduced in SP under both photopic (well-lit) and scotopic (dimly-lit) conditions at higher stimulus intensities, and using a flicker paradigm (further indicating impaired cone function) [8].

Furthermore, the variations in the dopamine (DA) activity that take place in the brains of SP also occur in their retinas. DA is the most essential neurotransmitter and modulator in the retina [3, 5]. It has been shown that DA precursor therapy is related to improvement in visual performance [15]. DA excess may enhance, whereas DA deficit may decrease color perception [3, 7, 11]. Therefore, the decrease in retinal DA causes reduced color vision and contrast sensitivity as well as lessened visual acuity [7]. Such retinal deformities and derangements may imply further abnormalities in the other constituents of the optical pathway. If the abnormal functioning begins at the retina, where coding of visual input is initiated, signaling at the subcortical and possibly cortical levels would be weaker [8, 12].

### Color discrimination

A study done by Shuwairi et al. showed that SP make more errors than controls in several measures of color discrimination, but do not display vulnerability specific to any hue axis. This is in contrast to findings of blue-hue axis (tritan) deficiencies in other conditions that involve dopaminergic depletion such as PD and cocaine withdrawal [10]. Another study carried out by Dahdouh et al. also demonstrated that patients with SCZ exhibit color discrimination deficits, not specific to any hue axis, compared to healthy controls [7]. These two studies did not find any correlation between antipsychotic medication dosage, assessed by the chlorpromazine equivalent doses, and color discrimination in SCZ. The results of the two studies conform to those reported by Haug et al. who demonstrated that psychiatric patients suffering from hypodopaminergia make color discrimination errors not specific to any axis and found no correlation between the dosage of antipsychotic medications and heterochromatic thresholds [7, 10, 16]. In contrast, Fernandes et al. indicated that color discrimination is impaired in SP, particularly in those taking typical antipsychotics, implying that antipsychotic medication may affect on color vision. But, this cannot be confirmed until a group of non-medicated patients is compared to that of medicated patients as well as identifying symptomatology via more comprehensive rating scales for the specific symptom categories [17].

While no correlation between color blindness and positive or negative SCZ symptoms was found by Shuwairi et al., Dahdouh et al. showed that color blindness was correlated to the severity of schizophrenic overall symptoms only in the subgroup of patients with severe SCZ, but no particular correlation was found with either positive or negative psychotic symptoms [7, 10]. Moreover, SP performed poorer than healthy controls in the color vision test used (Farnsworth D-15 test). Nevertheless, this study has several limitations. In fact, the poor performance in the Farnsworth D-15 test could have been a result of cognitive impairment and not of SCZ intrinsically. Therefore, it is imperative to perform an additional task to test cognition in SP, where this task should be similar to the Farnsworth D-15 test and of equal difficulty, but does not recruit any visual processes that are known to be impaired in SCZ and related to color processing. Additionally, color discrimination was assessed based on the total error score (TES), not axis-specific errors. Consequently, the color axis that is most deficient in SCZ could not be specified [7]. However, it is known that individuals with conditions (PD, cocaine withdrawal) that involve dopaminergic depletion display blue-hue (tritan) axis deficiencies. Given this and considering that SP suffer from dopaminergic dysregulations, hypodopaminergia being one of these dysregulations, it is of great advantage to assess hue discrimination in SCZ [10]. In the current study, we aimed to investigate the correlation between cognition and performance of SP on the color vision test, examine if color discrimination deficits are correlated to SCZ symptoms, and determine whether SP display discrimination deficits specific to a certain hue axis.

## Methods

### Participants

**Fig. 1:**
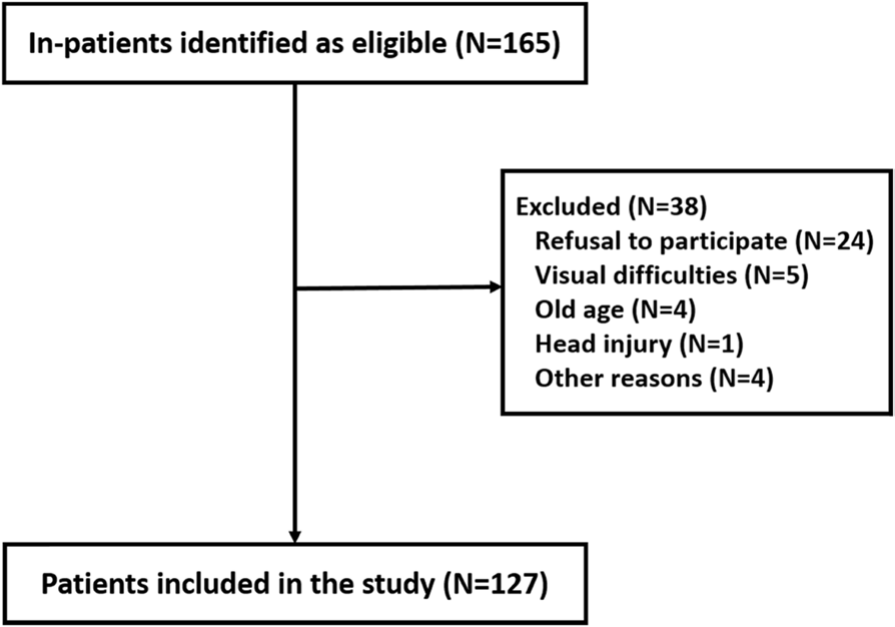
Flow diagram: patients included in the study

Participants included 127 SP recruited from the Psychiatric Hospital of the Cross (PHC). The SCZ in-patient database identified 165 in-patients as eligible for inclusion in the study, from whom 127 patients have met the inclusion criteria (Fig. 1). Those patients are chronic patients who have been hospitalized for more than a year. Patients included in the study are diagnosed with SCZ according to the Diagnostic and Statistical Manual of Mental Disorders, Fifth Edition (DSM*-5*) criteria, as indicated in their files, without any family history of color blindness, history of head injury, nor neurological or sensory problems. Excluded patients were those having neurological or sensory problems were excluded since those problems can cause the patient visual difficulties or affect the patient’s cognition adversely ([18] https://www.hopkinsmedicine.org/health/conditions-and-diseases/neurovisual-disorders), those abusing substances and alcohol, and having any visual difficulties or suffering from color.

### Procedures and assessments measurement

The measures used during the interviews were in Arabic, the native language of Lebanon. A questionnaire was used to determine the socio-demographic characteristics (age, gender, age at the first episode, the number of episodes, and family history of mental disorder). The presence and extent of schizophrenic symptoms were evaluated by the Positive and Negative Syndrome Scale (PANSS) scale. The Montreal Cognitive Assessment (MoCA) test was used to assess cognition, while the Farnsworth D-15 test was used to evaluate color discrimination. One trained person was responsible for the data collection, via a personal interview with each patient. No time limit was posed for the completion of the questionnaire as recommended for cognitively impaired individuals. Chlorpromazine equivalents of the neuroleptic medications that the patients were taking at the time of the study were calculated based on the method of Andreasen et al. [13].

### PANSS rating scale

The previously validated Arabic version of the PANSS was used [19]. The PANSS is a rating scale that consists of 30 items, basically organized into three different subscales: the positive symptoms (seven items P1 – P7), negative symptoms (seven items N1 – N7), and general psychopathology (16 items G 1 - G16). This scale was designed to assess the severity of psychopathology in adult patients with SCZ and other psychotic disorders [20]. Scoring of all items ranges from 1 to 7, with 1 signifying the absence of symptoms and 7 signifying extremely severe symptoms. By summing the score across each component item, we obtain a total score for each subscale, ranging from 7 to 49 for the Positive and Negative Scales and 16 to 112 for the General Psychopathology Scale [20].

### Farnsworth D-15 test

The Farnsworth D-15 test contains 15 color squares with a reference or pilot square. Participants being tested must rearrange the squares, placed in random order, in the correct color-coded order. The online version of the test (https://www.color-blindness.com/color-arrangement-test/) was used and a different initial random order was generated by the online system for each patient in an automatic process. Participants were instructed to arrange the 15 squares starting with the pilot, then choosing the most analogous square and dragging it to the allotted box. At the end of this test, each patient was distinguished as being not color- blind, slightly color-blind, moderately color-blind or severely color-blind depending on the TES that was generated for each patient. The higher the TES is, the more severely color-blind the patient is. For strong vision deficiencies, the TES ranges from around 11 up to about 40. Moreover, the deficient hue axis in color-blind people is also generated at the end of the test. The type of color blindness is quantified through a few scores, which are:

1. Confusion Angle: It identifies the type of color vision deficiency.
2. Major and Minor Radii: The ratio of those two numbers results in the Selectivity Index.
3. Total Error Score (TES): Combining the two radii into a score of total error.
4. Selectivity Index: This ratio shows the parallelism of the confusion vectors to the personal confusion angle.
5. Confusion Index: The ratio between the major radius and the major radius of a perfect arrangement.

The test was administered in binocular vision under true daylight to all participants.

### MoCA test

Validated in Lebanon [21], the MoCA test is a one-page 30 -point screening test designed to detect mild cognitive impairment (MCI). It includes the cognitive aspects of executive function and visuospatial abilities, naming, short-term memory, attention and working memory, language, concentration, verbal abstraction, and orientation. The total possible score is 30 reflecting no errors; a score of 26 or above is regarded as normal; and a score below 26 indicates MCI. An extra point is added if the individual has 12 years of education or less, to correct scores for low education [22].

### Statistical analysis

Data analysis was done using SPSS version 23 (statistical package for social science, SPSS, Chicago, IL). The TES score was normally distributed (skewness and kurtosis values between − 1 and + 1 [23]); The Description of the Farnsworth D-15 Dichotomous Color Blindness Test was performed using the counts and percentage. The Student t and ANOVA tests were used considering the TES score as the dependent variable to compare two and three or more means respectively. Pearson test was used to correlate two continuous variables. A linear regression taking the TES score as the dependent variable and the PANSS subscales, MoCA test, chlorpromazine equivalent dose, family history of psychiatric illness duration of hospitalization and illness as independent variables was performed. Also a multinomial regression (taking the type of blindness as the dependent variable and age, chlorpromazine equivalent dose, MoCA cognitive assessment test, duration of hospitalization, total PANSS score, gender and family history of psychiatric illness was performed**.** All the variables that showed a *p*-value < 0.2 in the bivariate analysis were included in the models to eliminate potential confounding factors [24]. Statistical significance was defined at *p* < 0.05.

## Results

### Sample description

Table 1 shows the demographic and other characteristics of patients with SZSCZ. The mean age of the patients was 56.09 ± 10.69 years, with 64.6% male. The majority (93.7%) were single, with a low education level (complementary level and below: 69.1%). Only 39.4% have a family history of psychiatric illness. The mean duration of illness and hospitalization in years was 28.53 ± 12.11 and 18.39 ± 10.56 respectively. The mean number of hospitalizations was 6.16 ± 6.54 and the mean chlorpromazine equivalent dose was 1037.76 ± 1008.89.

**Table 1:** Sociodemographic and other characteristics of the participants (*N* = 127)

Variable	N (%)
**Gender**	
Male	82 (64.6%)
Female	45 (35.4%)
**Marital status**	
Single/divorced/widowed	119 (93.7%)
Married	8 (6.3%)
**Education level**	
Illiterate	11 (8.7%)
Primary	39 (30.7%)
Complementary	37 (29.1%)
Secondary	27 (21.3%)
University	13 (10.2%)
**Family history of psychiatric illness**	
Yes	50 (39.4%)
No	77 (60.6%)
	**Mean ± SD**
**Age (in years)**	56.09 ± 10.69
**Duration of illness in years**	28.53 ± 12.11
**Duration of hospitalization in years**	18.39 ± 10.56
**Number of hospitalizations**	6.16 ± 6.54
**Chlorpromazine equivalent dose (mg)**	1037.76 ± 1008.89

### Description of the Farnsworth D-15 dichotomous color blindness test

Only 2.4% of the participants have severe color blindness and 21.3% have moderate color blindness. Protan color blindness has been found in 39.4% of the participants, 14.2% have deutan color blindness and 18.1% have tritan color blindness. The description of the color test is detailed in Table 2.

**Table 2:** Description of the Farnsworth D-15 Dichotomous Color Blindness Test

	N (%)
**Type of color blindness**	
None	36 (28.3%)
Protan	50 (39.4%)
Deutan	18 (14.2%)
Tritan	23 (18.1%)
**Severity of color blindness**	
None	36 (28.3%)
Slight	61 (48.0%)
Moderate	27 (21.3%)
Severe	3 (2.4%)
	**Mean**
**Confusion Angle**	19.94 ± 62.50
**Major Radius**	18.44 ± 5.70
**Minor Radius**	10.66 ± 4.27
**Total Score of Error**	21.45 ± 6.64
**Selectivity Index**	1.85 ± 0.60
**Confusion Index**	2.18 ± 2.33

### Bivariate analysis: correlates of the TES

Table 3 shows the bivariate analysis taking the TES as the dependent variable. The results showed that a higher cognition (*r* = − 0.318, *p* < 0.001) was significantly associated with a lower TES. Moreover, a higher duration of hospitalization (*r* = 0.190, *p* = 0.033), a higher PANSS score (*r* = 0.244, *p* = 0.006), a higher positive PANSS score (*r* = 0.255, *p* = 0.004) and a higher negative PANSS score (*r* = 0.208, *p* = 0.019) were significantly associated with a higher TES.

**Table 3:** Bivariate analysis taking the TES (Total Score of Error) as the dependent variable

	TES (Total Score of Error)	***F*** value and ***t***-value	***p***-value
**Gender**			
Male	21.62 ± 6.92	*t*-value: 0.376	0.708
Female	21.16 ± 6.16		
**Marital status**			
Single/divorced/widowed	21.44 ± 6.71	*t*-value: −0.094	0.925
Married	21.67 ± 5.73		
**Education level**			
Illiterate	23.46 ± 7.37	*F* value: 0.617	0.651
Primary	21.76 ± 6.46		
Complementary	21.72 ± 7.63		
Secondary	19.98 ± 5.12		
University	21.14 ± 6.65		
**Family history of psychiatric illness**			
Yes	20.33 ± 6.57	*t*-value: 1.554	0.123
No	22.19 ± 6.62		
	**Correlation coefficient**		***p*-value**
**Age**	0.102		0.252
**MoCA cognitive assessment test**	−0.318		**< 0.001**
**Duration of illness in years**	0.150		0.093
**Duration of hospitalization in years**	0.190		**0.033**
**Chlorpromazine equivalent dose**	0.162		0.069
**PANSS total scale**	0.244		**0.006**
Positive PANSS scale	0.255		**0.004**
Negative PANSS scale	0.208		**0.019**
General psychopathology PANSS scale	0.155		0.082

Numbers in bold indicate significant *p*-values

### Multivariable analysis

The linear regression results, taking the TES as the dependent variable and the PANSS subscales, MoCA test, chlorpromazine equivalent dose, family history of psychiatric illness duration of hospitalization and illness as the independent variables, showed that higher cognition (MoCA test) (Beta = −0.279) was significantly associated with a lower TES. Moreover, a higher positive PANSS score (Beta = 0.217) was significantly associated with a higher TES (Table 4).

**Table 4:** Multivariable analysis

Linear regression model taking the TES as the dependent variable and the PANSS subscales, MoCA test, chlorpromazine equivalent dose, family history of psychiatric illness duration of hospitalization and illness as independent variables.					
Variable	Unstandardized Beta	Standardized Beta	***P***	95% Confidence Interval	
**Family history of psychiatric illness**	−1.531	− 0.113	0.191	−3.834	0.772
**MoCA cognitive assessment test**	−0.279	− 0.262	**0.010**	− 0.491	− 0.067
**Duration of illness in years**	− 0.006	− 0.011	0.923	− 0.130	0.118
**Chlorpromazine equivalent dose**	0.001	0.106	0.217	−0.001	0.002
**Duration of hospitalization in years**	0.057	0.091	0.438	−0.089	0.203
**Positive PANSS scale**	0.217	0.240	**0.015**	0.044	0.391
**Negative PANSS scale**	0.103	0.113	0.271	−0.081	0.287
**General psychopathology PANSS scale**	−0.087	−0.158	0.185	−0.215	0.042

Numbers in bold indicate significant *p*-values

A third multinomial regression analysis taking the type of color blindness as the dependent variable showed being a female as compared to a male (ORa = 5.46) was significantly associated with more deutan type (Table 5). No significant association was found for the protan and tritan types.

**Table 5:** Multinomial logistic regression taking the type of color blindness as the dependent variable

	***P*** -value	ORa	95% Confidence Interval	
**Step 1: Protan vs none**^a^				
Age	0.253	0.969	0.917	1.023
Chlorpromazine equivalent dose	0.299	1.000	1.000	1.001
MoCA cognitive assessment test	0.068	0.924	0.849	1.006
Duration of hospitalization	0.179	1.041	0.982	1.104
Total PANSS score	0.585	1.008	0.980	1.036
Gender (Female vs Male^a^)	0.301	0.542	0.170	1.729
Family history of psychiatric illness (Yes vs No^a^)	0.280	0.586	0.222	1.544
**Step 2: Deutan vs none**^a^				
Age	0.667	0.985	0.918	1.056
Chlorpromazine equivalent dose	0.372	1.000	1.000	1.001
MoCA cognitive assessment test	0.833	0.987	0.877	1.111
Duration of hospitalization	0.923	0.996	0.921	1.078
Total PANSS score	0.149	1.024	0.991	1.059
Gender (Female vs Male^a^)	**0.024**	5.467	1.250	23.914
Family history of psychiatric illness (Yes vs No^a^)	0.570	1.451	0.401	5.254
**Step 3: Tritan vs none**^a^				
Age	0.448	1.026	0.960	1.098
Chlorpromazine equivalent dose	0.963	1.000	0.999	1.001
MoCA cognitive assessment test	0.475	0.963	0.868	1.068
Duration of hospitalization	0.437	1.026	0.961	1.096
Total PANSS score	0.857	1.003	0.969	1.038
Gender (Female vs Male^a^)	0.730	1.254	0.348	4.522
Family history of psychiatric illness (Yes vs No^a^)	0.076	0.320	0.091	1.125

^a^Reference group

## Discussion

SP in Shuwairi et al. did not make any errors in the Farnsworth D-15 test, while 91 patients in our study showed deficient performance on this test. The absence of errors in Shuwairi et al. may be due to the limited number of patients included (*n* = 16) [10].

Various studies showed that SP display poorer color discrimination than healthy individuals as the former group makes more errors in several tests of color vision [7, 10, 17]. Contradicting previous findings [10, 16], our study found those errors to be specific to the red-hue axis. This was discerned considering that protan color blindness is the most prevalent among color-blind SP in our study. In consistency with another study [10], subjects in our study did not exhibit differential vulnerability to tritan errors. A significant correlation between female sex and deutan color blindness was observed. In contrast to our results, Fakorede et al. reported that the percentage of males suffering from deutan colour blindness (2.33%) is higher than that of females suffering from this type of blindness (0.79%) [25]. Another study noted a greater prevalence of deutan and protan defects in males than in females [26].

When correlating the MoCA test score with the TES, we found a significant negative relationship. The higher the cognition is (higher MoCA score), the less severe the color discrimination deficits are (lower TES). This suggests that the poor performance of SP in the color discrimination test may be because of cognitive impairment and not SCZ itself. In contrast, a study done by Fernandes et al. reported poorer color performance in SP, excluding those suffering from MCI, than in healthy controls [17].

We found no significant correlation between the total PANSS score and the TES. These results differ from Dahdouh et al. which showed a positive correlation between overall symptoms and color blindness, but only in the subgroup of patients with severe SCZ [7]. Furthermore, some studies recounted a correlation of visual deficits with either overall symptoms [27, 28] or negative symptoms [29–33], but seldom with positive symptoms [34]. Our results revealed no significant correlation of TES with negative or general symptoms in concordance with other studies, but showed a significant positive correlation with positive symptoms, in contrast with previous studies [7, 10]. The more severe positive symptoms are (higher positive PANSS score), the more severe color discrimination impairment is (higher TES).

Positive symptoms in SCZ are caused by a state of hyperdopaminergia [1, 35, 36]. DA is a major neurotransmitter in the vertebrate retina. Data implies that the excesses and reductions in DA activity frequently occurring in the brains of SP are likely to also occur in the retina, where they may play an important role in visual perception disturbances [5]. Usually, hyperdopaminergia in the retina leads to heightened intensity of color [7]. However, increased DA release may be caused by the dysregulation of glutamate receptors. This dysregulation also induces increased Glu release which may be the reason behind the color discrimination impairment in our study [5]. If dopaminergic disruption, irrespective of high or low, causes impairment in color discrimination such as that observed in conditions characterized by hypodopaminergia (PD and cocaine withdrawal), SP would be anticipated to demonstrate an analogous pattern of performance. Particularly, they should significantly make more tritan errors than other types of errors [10]. Patients in our study made errors specific to the red-hue axis. Therefore, this suggests that DA dysregulation may not be the cause of color discrimination impairment, but rather supports our hypothesis of glutamatergic dysregulation being the cause. Nevertheless, we do not know if changes in Glu in the retina mirror those occurring in the brain (as in DA) in SCZ. Consequently, further research is necessary to investigate Glu and DA changes in the retina of individuals with SCZ.

No significant correlation exists between the antipsychotic medications, represented by the chlorpromazine equivalent doses, and TES, in accordance with other studies [7, 10, 16]. Nonetheless, the absence of correlation between color discrimination performance and antipsychotic medication dosage in SCZ does not exclude the effects of antipsychotic medication. In fact, a study done by Fernandes et al. reported poorer performance on color vision tests in SP taking typical antipsychotics compared to those taking atypical antipsychotics, as well as a positive correlation between the color vision test and the scale measuring schizophrenic symptoms. This suggested that deficient color discrimination in SCZ might be due to the effect of SCZ itself and/or antipsychotic medications. However, further studies are required where the performance of unmedicated patients should be compared to that of medicated patients [17]. Furthermore, antipsychotic agents can change cognitive performance that may modify visual performance. Moreover, in SP taking antipsychotic medication, blockage of DA receptors occurs causing visual deficits [7]. Still, the non-existence of such correlation, in addition to the lack of correlation between TES and the duration of illness provide further evidence for our hypothesis and calls into question the role of DA in color discrimination impairment.

### Clinical implications

Color vision impairment is a typical symptom of SCZ, and it can be more severe when combined with psychotic symptoms. Therefore, color vision in patients with SCZ has the potential to be used as a diagnostic and prognostic tool. Detecting color discrimination impairment could be a sign to consider, or an objective test to score, during the diagnosis of SCZ, and hopefully could be considered as a prognostic factor if further studies, comparing chronic SCZ patients with recent-onset SCZ, show that color discrimination impairment wanes during and after symptoms remissions and waxes during relapses. To illustrate, good color vision status would imply a good prognosis of SCZ and vice versa. Improvements or deteriorations in the prognosis of SCZ could be said to mirror improvements or deteriorations in color vision. Furthermore, this DA-visual-mental status correlation could be of major importance for clinical and paraclinical correlation studies. Future studies can investigate the correlation between DA and Glu neurotransmitters and color discrimination in SCZ. If such a correlation was established, then an indirect qualitative assessment of those neurotransmitters could be inferred from the color vision status.

Those implications are highly promising as they suggest that the eye can be a mirror of the brain, as the variations in the function of the eye would reflect those of the brain.

### Strengths and limitations

Our study included a sample of 127 patients, which is larger than any sample tested by any of the studies investigating color vision deficits in SCZ [7, 10, 17]. There are several limitations of this study. Endogenous dopaminergic dysregulation and DA-targeting medications were not evaluated in this study. In addition, the correlation between color discrimination and each individual antipsychotic medication is not separately investigated. Rather all antipsychotic medications taken by the patients were represented by the chlorpromazine equivalent dose. Due to the lack of funds, we did not use technical instruments for detecting retinal abnormalities. We also did not utilize brain imaging to identify cortical and subcortical impairments or assess cognitive impairment anatomically. Information bias is possible since patients might not understand a certain question or over/underrate it. There is also a possibility of a selection bias since the sample was recruited from a single institution. A residual confounding bias is also possible since not all factors associated with color discrimination were considered. Both the Panel D-15 and the 100-hue tests can differentiate between protan, deutan, and tritan defects. However, the Farnsworth Panel D-15 is a rapid test only of gross color confusions. The FM 100-hue test takes more time, which might be complicated for SP, but it is thought to be a more sensitive test for color discrimination. Additionally, a more comprehensive test could have been used to assess cognition instead of the MoCA test. However, although the MoCA test is brief with lower specificity than other more domain-specific tests, it is a simple test adequate for SP. At last, the absence of a control group constitutes another limitation of our study. Still, our work is based on the results of several previous studies, which all identified that SP make more errors than healthy controls [7, 10, 17].

## Conclusion

In summary, our results show a significant correlation of color discrimination deficits with low cognition as well as with the positive symptoms of SCZ. This suggests that color discrimination deficits, specific to the red-hue axis, may be due to the effect of cognitive impairment and/or SCZ itself. However, future work should assess DA and Glu changes in the retinas of SP and whether these changes contribute to visual functioning in SCZ. Prospective research can investigate the possibility of slight cognitive impairment affecting the performance of SP on color vision tests.

## Data Availability

All data generated or analyzed during this study are not publicly available due to restrictions from the ethics committee. The dataset supporting the conclusions is available upon request to the corresponding author.
